# Risk perception of COVID-19 and its related factors among centralized medical isolation groups in China

**DOI:** 10.3389/fpsyg.2023.1131076

**Published:** 2023-02-01

**Authors:** Rui Zhang, Chengli Wang

**Affiliations:** School of Public Policy & Management and School of Emergency Management, China University of Mining and Technology, Xuzhou, China

**Keywords:** COVID-19, risk perception, centralized medical isolation, psychological health, cognitive-experiential self-theory (CEST), common sense model of risk perception (CSM)

## Abstract

**Objective:**

Medical isolation is one of the most effective measures to slow the spread of the virus when dealing with a pandemic. Millions of people in China have undergone centralized medical isolation (CMI) during the COVID-19 pandemic. This study aims to assess the centralized medical isolation group’s COVID-19 risk perception and to explore the influencing factors.

**Methods:**

A total of 400 participants (200 who had experienced CMI and 200 who had not experienced) completed a questionnaire related to COVID-19 risk perceptions. The questionnaire was designed with the Cognitive-Experiential Self-Theory (CEST) and the Common Sense Model of Risk Perception (CSM). It adopted nine questions to measure risk perception in terms of Emotional feelings, Cognitive judgment, and Mental representation of unusual severity. Descriptive statistical analysis, correlation analysis, and multiple linear regression analysis were conducted with SPSS 26.0 software.

**Results:**

The mean risk perception score for the CMI group was 30.75, with a standard deviation of 7.503, which was significantly higher than that in the non-centralized medical isolation (NCMI) group (risk perception score was 28.2, and the standard deviation was 7.129). The results show that risk perceptions were higher for older age, risk perceptions were higher for higher education, risk perceptions were higher for those who had received the COVID-19 vaccination, and risk perceptions were higher for those who lived in a family with children.

**Conclusion:**

Risk perception is significantly higher in CMI groups than in NCMI groups. The government should draw more care to the risk perception and psychological wellbeing of the CMI group and provide extra support and assistance to the elderly and those raising younger children. In dealing with future pandemics like the COVID-19 outbreak, the government should actively guide the public to properly isolate at home and cautiously implement a CMI policy.

## 1. Introduction

Since the end of 2019, the COVID-19 outbreak has become a severe global pandemic affecting people’s daily lives and economic activities. The COVID-19 pandemic is still not effectively controlled in some countries. However, an increasing number of countries are reducing their precautionary measures and seeking to enter a phase of normalization where they can coexist with COVID-19. China is gradually relaxing its control of COVID-19, mainly by releasing restrictions on the movement of people across regions, replacing the mandatory centralized medical isolation (CMI) of confirmed cases with voluntary centralized medical or home isolation, and abolishing the checking of negative nucleic acid certificates from the public in the vast majority of premises.

Medical isolation is a measure of isolation for people who are carriers of a specific virus or have developed a specific disease (Jiang et al., 2022). During the COVID-19 pandemic, many countries worldwide implemented medical isolation policies. Most countries had required people who tested positive for nucleic acid for COVID-19 to be quarantined at home for 5–10 days, which could end on its own when the nucleic acid test turned negative. According to an Agence France-Presse (AFP) report on 29 March 2020, more than 3.38 billion people worldwide were required to comply with medical isolation measures to fight COVID-19. This figure represents about 43% of the global population^1^. China has adopted more stringent medical isolation measures than most countries. China has CMI for confirmed cases, suspected cases, close contacts of asymptomatic infected people, and people entering the country, requiring them to undergo daily morning and evening temperature and health status monitoring and regular nucleic acid collection. There are two types of CMI in China, one for hotel isolation (mainly for entry personnel and close contacts of COVID-19 confirmed cases) and one for square-cabin hospital isolation (mainly for COVID-19 lightly infected patients, asymptomatic infected patients, and a few close contacts). According to official data released by China, 189,669 people were still under medical isolation and observation related to COVID-19 in China as of 19 November 2022^2^.

Although the risk posed by COVID-19 to public health is decreasing, there are still many people whose risk perceptions have been or are being affected. *Risk perceptions* are people’s beliefs, attitudes, judgments and emotions about risks (Slovic, 1987; Zhang Y. B. et al., 2020). In response to a pandemic like the COVID-19 outbreak, the public’s willingness and behavior to maintain social distance, voluntarily vaccinate, observe travel restrictions, and comply with quarantine measures can slow the spread of the pandemic and accelerate the recovery of the public health system (Brewer et al., 2007; Ferrer and Klein, 2015; Wise et al., 2020; Zhang et al., 2023). When the public’s risk perception is high, they will adopt stricter preventive behaviors and are more inclined to comply with government pandemic prevention measures. When the public’s risk perception is low, they will adopt lower levels of preventive behavior and may even fight against some of the prevention measures (Weinstein and Nicolich, 1993; Taghrir et al., 2020). Multiple factors can influence the public’s perception of risk. Luo et al. (2021) conducted a literature review based on international databases and showed that isolation, mental disorders, and restricted social activities due to blockades increased the fear of COVID-19. The mean fear of COVID-19 was higher in women than in men (20.67 vs. 18.21). Females in Asia (18.36) and Australia (17.43) had the highest mean and lowest mean fear of COVID-19, and females in hospital staff (19.51) and university students (17.95) had the lowest mean fear. Several studies have been conducted to demonstrate the impact of home isolation and social distance on public perceptions of risk during the COVID-19 outbreak. The study conducted by Parisi et al. (2021) showed that women in Italy tended to go out less and take personal protective measures during the COVID-19 pandemic. The resulting physical distance and housing isolation exacerbated family conflicts and affected community neighborhoods. Zhang M. M. et al. (2020) found that home isolation caused many negative emotional reactions in nurses and that the nurses’ positive emotions gradually increased as the isolation period ended. Abu Jamileh et al. (2021) conducted a cross-sectional study in Saudi Arabia with parents of children aged 6–14 years, and found that children experienced mild-to-severe psychological impact on behaviors and feelings during home isolation during COVID-19 pandemic. Denerel et al. (2022) found that home isolation affected the mental health of athletes, although this effect was weaker compared to non-athletes. Slone et al. (2022) found that home isolation caused psychological distress in the public and that the severity of this psychological distress was inversely related to the public’s ability to exercise Self-Mastery (Self-Mastery refers to a sense of having control over life events and is reflected in a self-perception of strength and the capacity to cope with and overcome obstacles by relying on personal efforts). Shechory and Laufer surveyed Israelis living in conflict zones. They found that respondents feared COVID-19 more than terrorism during Israel’s enforced blockade. These respondents living in conflict areas did not show higher levels of resilience when faced with new threats during the COVID-19 pandemic. However, they may have been more adept at dealing with long-standing threats, such as terrorism (Shechory, and Laufer, 2021). The results of a study conducted by Rania and Coppola in Italy, which lasted 14 days, showed that fear of COVID-19 was an emotional state experienced by the population as a whole and that there was a positive correlation between COVID-19-related fear, feelings of isolation, mental health and a positive sense of social distance. Younger people were likelier to be lonely and less likely to accept the imposed social distance than older people (Rania and Coppola, 2022). Ju et al. (2021) assessed the psychological state of the COVID-19 patients and found that the form and duration of isolation significantly affected levels of depression and anxiety, with home isolated patients experiencing significantly less depression and anxiety, compared to centrally isolated patients who did not. Although some scholars have focused on the home isolation group, few have studied the risk perception of the CMI group.

Medical isolation effectively slowed the spread of the virus in the early stages of countries’ response to COVID-19, and it is still likely to be seen as one of the core measures to deal with future pandemics. Therefore, it is essential to assess and analyze the risk perceptions of CMI groups in order for governments to optimize medical isolation policies and better respond to public health emergencies. This study will assess the COVID-19 risk perceptions of CMI groups and analyze the factors influencing them based on research data. On this basis, we will compare the risk perceptions of CMI groups with those of non-centralized medical isolation (NCMI) groups to analyze better and demonstrate the impact of centralized isolation measures on public risk perception. This study will contribute to the development of risk perception research and provide a reference for the government to optimize its policy on CMI.

## 2. Materials and methods

### 2.1. Participants and procedures

Participants included 200 respondents undergoing CMI and 200 who had not experienced CMI. The study was conducted only for the public over 18, and the questionnaires completed by the participants were anonymous. The study was approved by the Ethics Committee of the School of Public Policy and Management of the China University of Mining and Technology (approved 6-2022). This survey was conducted by the researchers from China University of Mining and Technology. All respondents completed the questionnaire voluntarily. The respondents had freedom not to do so and could withdraw their consent during the research.

Centralized medical isolation groups (CMI groups): The researchers randomly selected five CMI sites in Jiangsu Province, China. After consultation and communication with the staff in charge of the isolation sites, the researchers pushed an electronic version of the questionnaire to the isolated people and collected the data. The researchers set up the questionnaire to be filled in only once by the same account to prevent duplication.

Non-centralized medical isolation groups (NCMI groups): The research site was a city in Jiangsu Province. The researchers used a multi-stage stratified random sampling method. The questionnaires were distributed and collected face-to-face. The researchers verbally asked the respondents if they had experienced CMI before distributing the questionnaire, and if they had, they would not be selected as respondents.

### 2.2. Questionnaire

The COVID-19 pandemic has the characteristics of a massive stressor (intimidating, prolonged, unpredictable, and severe consequences). These characteristics of COVID-19 inevitably affect the risk perception for individuals and the general public. Existing risk perception measurement studies may provide a framework for insight into the assessment of risk perception of the COVID-19 pandemic in this study. Slovic (1987) proposed a psychometric model to assess risk perception characteristics, mainly regarding risk familiarity and controllability dimensions. Shi et al. (2003) used the model proposed by Slovic to assess the public’s risk perception of severe acute respiratory syndrome coronavirus (SARS) information, and some valuable evidence was obtained. However, this assessment is *ex post*-oriented, with minimal applicability during the outbreak. Xie et al. (2005) developed a three-dimensional, 11-item SARS risk perception assessment tool based on Slovic’s model, including apprehensiveness, controllability, and likelihood of infection. This tool has the advantage of assessing the risk perception of the outbreak/virus from three dimensions but still has the disadvantage of simplifying the risk perception.

This study referred to the above studies and designed a questionnaire based on the Cognitive-Experiential Self-Theory (CEST) and the Common Sense Model of Risk Perception (CSM). The basic assumption of CEST is that humans respond through both empirical and rational systems (Pacini et al., 1998). The empirical system is primarily non-verbal and imaginative and learns automatically from experience; the rational system is the human-specific verbal reasoning system. The failure of one of these two systems can lead to adaptation problems for the individual. For example, there is a discrepancy between knowledge and action: the risk of not wearing a mask despite knowing the risk of infection is not based on a genuine fear of the new coronavirus. Another example is the lack of scientific knowledge about the NIV when one is so afraid that one cannot leave the house even when the pandemic has improved. When both failures occur, individuals exhibit more severe maladjustment, which may explain the wide range of reactions and behaviors during a pandemic. The CEST illustrates the need to balance perceptions and feelings in measuring public perceptions of risk in significant public health emergencies.

The CSM is a model of health threat self-regulation developed by Leventhal et al. (1998) The model suggests that health threat information activates and develops a risk representation of the disease, which includes identification, etiology, timeline, consequences, and cure. Individuals, groups, and societies use risk representations to move toward selecting and using health threat control procedures. Specifically, risk representations include two intertwined components that are difficult to separate: the representation of the disease/threat/risk and the emotional response. Emotions are infused into cognitive processes, influencing cognitive reasoning and distillation. The final response of the individual as a problem solver does not depend solely on the cognitive representation or the emotional response but rather on the final risk management decision as a result of the integration of the two.

In addition, we referenced the Perceived Risk of HIV Scale (PRHS) developed by Lucy et al. (2012) to provide a more operational sample reference for item writing for this study. Ultimately, we measured respondents’ COVID-19 risk perceptions in the questionnaire with three variables. These variables include the individual’s emotional feelings about their infection with COVID-19, their cognitive judgment of the vulnerability to infection, and the mental representation of unusual severity. Specifically, emotional feelings about one’s infection reflected a range of emotions, such as worry, fear, and even dread (Cui et al., 2021). The judgment of vulnerability refers to the respondent’s perception of how likely they are to be infected with COVID-19 (Capone et al., 2021). The mental representation of unusual severity reflects the level of alertness to the risk of COVID-19 and is a mixture of perceptions and feelings (Xi et al., 2020). The specific items set in the questionnaire are shown in Table 1.

**TABLE 1:** Questionnaire items designed based on Cognitive-Experiential Self-Theory (CEST) and Common Sense Model of Risk Perception (CSM).

Variables	Connotations	Item	Theoretical sources
Emotional feelings	Emotional response to the infection of COVID-19 (Zhong et al., 2021)	Item 6 How likely do you think you are to contract COVID-19?	CEST Intuitive feelings
		Item 7 Are you worried about contracting COVID-19?	CEST Intuitive feelings
		Item 8 Do you think you are susceptible to COVID-19?	CEST Intuitive feelings
Cognitive judgment	Judgment of vulnerability to infection (Zanin et al., 2020)	Item 9 Are you sure you won’t infect with COVID-19?	CEST Cognitive judgment
		Item 10 Do you think that you are at risk of contracting COVID-19, no matter how small the probability is?	CEST Cognitive judgment
		Item 11 What do you think your probability is of contracting COVID-19?	CEST Cognitive judgment
Mental representation of unusual severity	Constantly reminded of COVID-19 (Kuang et al., 2020)	Item 12 Is it hard for you to imagine yourself infected with COVID-19?	CSM Mental representation
		Item 13 Have you ever assumed you were infected with COVID-19?	CSM Mental representation
		Item 14 Have you ever assumed that a family member has been infected with COVID-19?	CSM Mental representation

After validating the questionnaire with the Delphi method, a questionnaire containing four sections was designed for this study. The first part was respondent characteristics, including gender, age, whether or not they had received the COVID-19 vaccination, whether or not they live in a family with children, and education. The second part is about emotional feelings. The third part is about cognitive judgments. The fourth part is about the mental representation of unusual severity. Parts 2, 3, and 4 are scored on a five-point scale, totaling 45 points for the nine questions. Higher scores represent a higher risk perception among the respondents.

### 2.3. Statistical analysis

This study adopted SPSS 26.0 for statistical analysis. Descriptive statistical analysis, reliability analysis, validity analysis, correlation analysis, and multiple linear regression analysis were conducted for this study. Frequency and composition ratios were used to describe the general information. Means and standard deviations were used to describe the scores, and the questionnaire scores were tested for normality. One-way ANOVA was used to compare the differences in the COVID-19 risk perceptions among people with different characteristics. Multiple linear regression analysis was applied to explore the factors influencing the COVID-19 risk perceptions.

## 3. Results

### 3.1. Study overview

In this study, 400 questionnaires were distributed and returned, of which 380 were valid, and the effective response rate of the questionnaires was 95%. The Effect Size for the data in this study was taken as the Pearson linear correlation coefficient between the variables (x1-x2, x1-x3,., x8-x9 with 9 questions as variables), with a significance level of 0.01, Power is taken as 0.9, and a two-tailed test is performed. The required sample size calculated from the *t*-test in Python is at least 210. Therefore, a sample size of 380 to meet the needs of this study. The Kaiser-Meyer-Olkin (KMO) test for the data was 0.923, which was greater than 0.9. The Bartlett test was 0, which was less than 0.05. The Cronbach Alpha result was 0.81, which was greater than 0.7. The above tests proved that the data had high reliability and validity and were suitable for analysis. Of the 380 valid questionnaires, 193 were from the CMI group, and 187 were from the NCMI group. The basic information of the respondents is shown in Table 2.

**TABLE 2:** Basic information of respondents.

Centralized isolation				No centralized isolation			
		**Number**	**%**			**Number**	**%**
Gender	Male	104	53.9	Gender	Male	88	47.1
	Female	89	46.1		Female	99	52.9
Age	18–28	47	24.3	Age	18–28	52	27.8
	29–40	93	48.2		29–40	59	31.5
	41–65	44	22.8		41–65	54	28.9
	66 and above	9	4.7		66 and above	22	11.8
Have you received the COVID-19 vaccination?	Yes	170	88.1	Have you received the COVID-19 vaccination?	Yes	161	86.1
	No	23	11.9		No	26	13.9
Do you live in a family with children?	Yes	105	54.4	Do you live in a family with children?	Yes	134	71.7
	No	88	45.6		No	53	28.3
Education	High School and below	57	29.5	Education	High School and below	62	33.1
	Bachelor	116	60.1		Bachelor	111	59.4
	Master and above	20	10.4		Master and above	14	7.5

### 3.2. COVID-19 risk perceptions among the CMI group

The mean risk perception score for the CMI group was 30.75, with a standard deviation of 7.503. Regarding the emotional feelings, 15.5% of the CMI group felt they were very likely to contract COVID-19, 14% were always worried about contracting COVID-19, and 12.4% felt they were highly vulnerable to contracting COVID-19. Regarding the cognitive judgment, only 4.7% of the CMI group were confident that they would not contract COVID-19, 20.2% strongly believed they were at risk of contracting COVID-19 regardless of the odds, and 16.6% believed that they would inevitably contract COVID-19. Regarding the mental representation of unusual severity, only 6.2% of the CMI group felt they could not imagine themselves with a COVID-19 infection, 18.7% always assumed that they had been infected with COVID-19, and 21.2% always assumed that a family member had been infected with COVID-19. The full results for the centrally isolated group are shown in Appendix A.

Pearson, Kendall, and Spearman correlations were performed to test different characteristics of the CMI group with risk perception scores, and all showed that COVID-19 vaccination, age, whether living in a family with children, education and risk perception scores were significantly correlated, while gender and risk perception scores were not significantly correlated (The correlation analysis results are available in Table 3).

**TABLE 3:** Correlation test results.

Test methods	Item		Gender	COVID-19 vaccination	Age	Do you live in a family with children?	Education
Pearson test	Risk perception score	Correlation index	-0.087	−0.607**	0.581**	−0.701**	0.147*
		Sig. (2-tailed)	0.227	0	0	0	0.041
Kendall’s Tau-b	Risk perception score	Correlation index	-0.059	−0.497**	0.469**	−0.604**	0.168**
		Sig. (2-tailed)	0.331	0	0	0	0.004
Spearman Rho	Risk perception score	Correlation index	-0.07	−0.595**	0.578**	−0.723**	0.204**
		Sig. (2-tailed)	0.332	0	0	0	0.004
			193	193	193	193	193

*Significant correlation at 0.05 level (2-tailed.)

**Significant correlation at 0.01 level (2-tailed).

We conducted a multiple linear regression analysis to analyze further the relationship between different characteristics of the CMI group and risk perceptions. We set gender, COVID-19 vaccination, age, whether living in a family with children and education as independent variables and risk perception scores as dependent variables. The *F*-value was 56.344, and the *p*-value was less than 0.01, indicating that the regression model was statistically significant (ANOVA test results are available in Table 4).

**TABLE 4:** ANOVA^a^ test results.

	Quadratic sum	Degree of freedom	Mean square	F	Sig.
Regression	6,497.269	5	1,299.454	56.344	0.000^b^
Residuals	4,312.793	187	23.063		
Total	10,810.062	192			

^a^Dependent variable: Risk perception score.

^b^Predictor variables: (Constants), gender, COVID-19 vaccination, age, do you live in a family with children, education.

Then, we conducted a multiple linear regression analysis (The results are available in Table 5). All five independent variables had a *p*-value of less than 0.05 in the regression model, indicating that they all had a regression effect on the dependent variable. The gender variable was not correlated with the risk perception score, but a regression effect relationship existed. According to the usual standard and existing research practices, we believe that the gender variable was not correlated with risk perception scores (Li and Chen, 2010; Sheng et al., 2022; Xiong et al., 2022). Therefore, we can get the following results in the CMI group: risk perceptions were higher for older age (*p* < 0.01); risk perceptions were higher for higher education (*p* < 0.01); risk perceptions were higher for those who had received the COVID-19 vaccination (*p* < 0.05); and risk perceptions were higher for those who lived in a family with children (*p* < 0.01).

**TABLE 5:** Regression model coefficient^a^ [the centralized medical isolation (CMI) group].

	Unstandardized coefficients		Standardized regressive coefficient beta	t	Sig.	VIF
	**B**	**Std. error**				
(Constants)	40.449	2.71		14.925	0.000	
Gender	-3.260	0.719	-0.217	-4.536	0.000	1.074
Age	2.512	0.522	0.271	4.812	0.000	1.487
COVID-19 Vaccination	-2.482	1.164	-0.156	-2.133	0.034	2.512
Living in a family with children	-6.669	1.169	-0.444	-5.704	0.000	2.838
Education	1.583	0.597	0.127	2.651	0.009	1.081

^a^Dependent variable: Risk perception score.

### 3.3. Comparative analysis of COVID-19 risk perceptions among the CMI and NCMI groups

**FIGURE 1:**
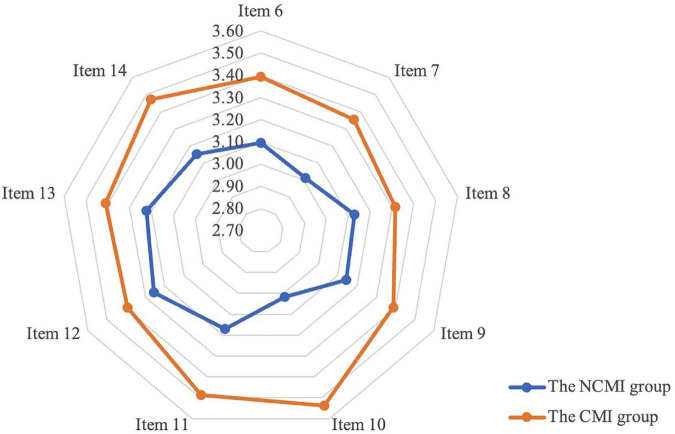
Comparison of risk perception scores between the centralized medical isolation (CMI) group and the non-centralized medical isolation (NCMI) group.

The average risk perception score for the non-centralized medical isolation (UCMI) group was 28.2 with a standard deviation of 7.129, significantly lower than that of the CMI group (average risk perception score of 30.75 with a standard deviation of 7.503). As shown in Figure 1, we calculated mean scores for each of the nine items measuring risk perception. We found that all scores for the risk perception component of the NCMI group were lower than those of the CMI group. This comparison strongly suggests that experiencing CMI increases the public’s perception of risk.

In order to compare the differences in the influencing factors between the CMI group and the NCMI group, correlation and regression analyses were conducted on the risk perception data from the NCMI group (the analysis results are available in Table 6). The results show that gender, COVID-19 vaccination and education did not have a statistically significant regression effect relationship on risk perception scores. Meanwhile, risk perceptions were higher for older age (*p* < 0.01) and higher for those living in a family with children (*p* < 0.01). Compared to the CMI group, there was no significant effect of COVID-19 vaccination and education on the risk perceptions of the NCMI group. Such results also suggest that age and living in a family with children significantly affected the public’s perception of risk, regardless of whether they had experienced CMI or not.

**TABLE 6:** Regression model coefficient^a^ [the non-centralized medical isolation (NCMI) group].

	Unstandardized coefficients		Standardized regressive coefficient beta	t	Sig.	VIF
	**B**	**Std. error**				
(Constants)	30.806	2.704		11.393	0.000	
Gender	1.121	0.658	0.079	1.704	0.09	1.033
Age	2.748	0.467	0.382	5.885	0.000	2.04
COVID-19 Vaccination	-2.684	1.711	-0.161	-1.569	0.118	5.124
Living in a family with children	-4.968	1.671	-0.315	-2.972	0.003	5.433
Education	-0.453	0.609	-0.037	-0.744	0.458	1.21

^a^Dependent variable: Risk perception score.

## 4. Discussion

### 4.1. Risk perceptions of COVID-19 in the CMI group

The mean risk perception score for the CMI group was 30.75 (out of a total of 45), with a standard deviation of 7.503, placing the risk perception at a high level. After correlation analysis, multiple regression analysis, and comparative analysis, we found that four factors influenced the risk perception of the CMI group.

Firstly, risk perceptions were higher for older age (*p* < 0.01). The average age of cases of death due to COVID-19 (direct effect or complication) reported from various countries is above 70 years. Public data from the centers for disease control and prevention (CDC) in the USA also show a higher risk of hospitalization and mortality in the older age group following the COVID-19 infection^3^. Thus, the older age group’s COVID-19 risk perceptions are higher than that of the younger age group (Batsis et al., 2021).

Secondly, risk perceptions were higher for those who had received the COVID-19 vaccination (*p* < 0.05). Protective behaviors of the public were strongly related to risk perception. When the public have a higher perception of risk, they will have the tendency to adopt protective behaviors to protect themselves (Abdelrahman, 2020; de Bruin and Bennett, 2020). Therefore, groups who had received the COVID-19 vaccination tended to have higher risk perceptions.

Thirdly, risk perceptions were higher for higher education (*p* < 0.01). More educated people generally had a higher willingness and ability to obtain information, and receiving more information increased their risk perception (Yu et al., 2020). The more educated group typically had relatively lower perceived severity and higher levels of COVID-19 apprehension compared to the less educated group (Rattay et al., 2021).

Fourth, the risk perception of living in a family with children was higher (*p* < 0.01). Although children are less likely than adults to develop critical illness from COVID-19, COVID-19 is a significant threat to children with underlying medical conditions (Dong et al., 2020). Infection with COVID-19 has an incalculable psychological impact on children and a potential risk of sequelae (Spinelli et al., 2020). People living in families with children are concerned about their children becoming infected with COVID-19, and they must put the extra effort into protecting their children from COVID-19. Furthermore, CMI is a totally closed process during which no relatives are allowed to visit. Groups with children in CMI may be concerned that their children are not better cared for at home, which is one of the key factors contributing to their perception of higher risk. Therefore, the risk perception of COVID-19 is higher among those living in families with children.

The level of COVID-19 risk perceptions was higher in the CMI group compared to the NCMI group. Age and living in a family with children had a significant effect on the COVID-19 risk perceptions in both the CMI groups and the NCMI groups. The COVID-19 vaccination and education did not have a significant effect on the COVID-19 risk perceptions in NCMI groups compared to CMI groups. These results suggest that the public is more concerned about the impact of COVID-19 on the elderly and children, regardless of whether they are in CMI. The CMI groups are people who test positive for nucleic acid for COVID-19 or are close contacts of confirmed cases and they have a greater probability to be detected as infected with COVID-19 (Ju et al., 2021). In addition, the CMI groups are subject to strict epidemiological standards during isolation and are restricted in their freedom for short periods (about 7 days). These measures further increase the psychological stress of the CMI groups and significantly increase their perception of risk.

### 4.2. Comparison with existing studies

Among the studies conducted, the study by Alessia et al. (2021) and He et al. (2021) showed an effect of age on the COVID-19 risk perception, with the older the group, the higher the perceived severity of COVID-19. The study results of Ding et al. (2020) and Ning et al. (2020) showed higher risk perception in the highly educated group. The study by He et al. (2021), Yan et al. (2021), and Cao et al. (2022) demonstrated higher risk perception in groups living in a family with children. Our results are consistent with the above studies. In contrast to these studies, we set the question “Have you ever received the COVID-19 vaccination.” Most of the studies that have been done on “risk perception” and “vaccination” have discussed what factors influence people’s perception of the risk of vaccines. However, few studies have captured the perceived risk status of vaccinated and unvaccinated groups. This study compares this and demonstrates that risk perceptions are higher among vaccinated groups, which is essential to studies on risk perceptions of COVID-19 and public self-protection behaviors. The conclusions drawn from this comparative analysis are more objective and scientific than those from studies of a single group.

### 4.3. Implications for policy, practice, and research

In the COVID-19 pandemic, CMI policies were implemented mainly in mainland China. For countries that have implemented or are likely to implement CMI policies in similar pandemics in the future, it is crucial to focus on the CMI groups.

Firstly, governments should be cautious in their choice of CMI policies. This study has demonstrated a higher level of risk perception among the CMI groups compared to the NCMI groups. Considering that medical isolation will remain one of the essential measures in dealing with similar pandemics in the future, governments should adopt a more humane approach to medical isolation policies (Zhang, 2020). For example, governments should take the initiative to guide the public toward proper home isolation, and provide CMI and the necessary support for those who do not have access to home isolation.

Secondly, in practice, the government should pay more attention to the CMI groups. Considering the negative psychological impact of CMI, the government should give the CMI groups more care and attention to their psychological health during the isolation period (Wang et al., 2021). The government may consider online interventions for the public during their intensive medical isolation to prevent psychological discomfort and mental depression. At the end of the isolation period, the government should provide free psychological health services to those who need them (Li et al., 2021). For older people and groups raising younger children, the government should provide them with additional help and support.

Finally, this study took the CMI groups as the study object. It demonstrated that the CMI groups had a higher perception of risk than the NCMI groups. Our results have allowed scholars to focus more on the risk perceptions and psychological health issues of the CMI groups and have provided a basis for further research. The comparative analysis of the CMI and NCMI groups in this study also provides a perspective for further research.

### 4.4. Study strengths and limitations

This study focuses on the CMI groups in the COVID-19 pandemic, which is one of the strengths. Government and scholars have ignored the COVID-19 risk perceptions in the CMI population. There are no government policies in place to address the psychological and mental health recovery of CMI groups, and few scholars have conducted research on the risk perceptions of CMI groups. However, at least millions of people in China have experienced CMI, and these people’s risk perceptions and psychological health status deserve attention and study. Moreover, medical isolation is an effective means of slowing down the spread of the virus. Therefore, this study’s analysis of risk perceptions of the CMI groups can provide a reference for the government when developing isolation policies in response to similar pandemics in the future. Another strength of this study is that it is a comparative study, which makes the findings more convincing. This study examined both the CMI and NCMI groups. This allowed for an analysis of the risk perceptions of the CMI groups and the factors influencing them, as well as a comparison of their risk perceptions with those of the NCMI groups.

There are some limitations in this study. Ideally, collecting as much basic information about the respondents as possible would have enabled a more accurate analysis of the factors influencing risk perception. However, due to concerns about influencing respondents’ willingness to answer questions, some specific basic information was not collected in this study (presence of accident and commercial insurance in the family, personal income, and presence of chronic diseases). In addition, the length of CMI has a potential impact on the public’s perception of risk, but we did not consider this in the questionnaire, which is a limitation of this study and one of the directions for further research.

## 5. Conclusion

This study showed that the COVID-19 risk perceptions was higher in the CMI groups compared to the NCMI groups. Age, COVID-19 vaccination, living in a family with children, and education significantly influenced risk perception in the CMI groups. In the future, scholars can further explore the relationship between income, insurance, chronic illness, and COVID-19 risk perception, particularly the impact of the length of CMI on public risk perception. Countries worldwide may adopt various isolation policies in respond to future pandemics like the COVID-19 outbreak, including CMI. Therefore, governments should pay more attention to the CMI groups to protect their physical health, and ensure their psychological and mental wellbeing. The public is more hopeful that the government has absorbed all the policy lessons learned and that future public health emergencies will not disrupt lives, livelihoods, and wellbeing on the same scale at which COVID-19 did.

## Data availability statement

The raw data supporting the conclusions of this article will be made available by the authors, without undue reservation.

## Ethics statement

This study was approved by the Ethics Committee of the School of Public Policy and Management of the China University of Mining and Technology (approved 6-2022). This study does not involve human or animal experiments.

## Author contributions

RZ: conceptualization, methodology, formal analysis, and writing—original draft. RZ and CW: investigation and writing—review and editing. Both authors read and agreed to the published version of the manuscript.

## Footnotes

1
https://baijiahao.baidu.com/s?id=1662757736370578679&wfr=spider&for=pc

2
https://m.thepaper.cn/baijiahao_20821188

3
https://www.cdc.gov/coronavirus/2019-ncov/covid-data/investigations-discovery/hospitalization-death-by-age.html

## Appendix A

**Table:** Questionnaire results for the centralized medical isolation (CMI) groups.

Item		Number	%	Item		Number	%
How likely do you think you are to contract COVID-19?	Extremely unlikely	13	6.7	What do you think your probability is of contracting COVID-19?	Never	9	4.7
	Unlikely	26	13.5		Low	26	13.5
	Medium	56	29		Medium	52	26.9
	Likely	68	35.2		High	74	38.3
	Extremely likely	30	15.6		Inevitable	32	16.6
Are you worried about contracting COVID-19?	Never	10	5.2	Is it hard for you to imagine yourself infected with COVID-19?	Extremely agree	12	6.2
	Seldom	31	16		Agree	27	14
	Medium	60	31.1		Medium	61	31.6
	Often	65	33.7		Disagree	59	30.6
	Always	27	14		Extremely disagree	34	17.6
Do you think you are susceptible to COVID-19?	Extremely disagree	9	4.7	Have you ever assumed you were infected with COVID-19?	Never	15	7.8
	Disagree	30	15.5		Seldom	31	16.1
	Medium	69	35.8		Medium	43	22.3
	Agree	61	31.6		Often	68	35.2
	Extremely agree	24	12.4		Always	36	18.6
Are you sure you won’t infect with COVID-19?	Extremely agree	9	4.7	Have you ever assumed that a family member has been infected with COVID-19?	Never	14	7.3
	Agree	32	16.6		Seldom	21	10.9
	Medium	60	31.1		Medium	59	30.5
	Disagree	59	30.5		Often	58	30.1
	Extremely disagree	33	17.1		Always	41	21.2
Do you think that you are at risk of contracting COVID-19, no matter how small the probability is?	Extremely disagree	8	4.2				
	Disagree	29	15				
	Medium	46	23.8				
	Agree	71	36.8				
	Extremely agree	39	20.2				
